# Association Mapping Provides Insights into the Origin and the Fine Structure of the Sorghum Aluminum Tolerance Locus, *Alt_SB_*

**DOI:** 10.1371/journal.pone.0087438

**Published:** 2014-01-30

**Authors:** Fernanda F. Caniato, Martha T. Hamblin, Claudia T. Guimaraes, Zhiwu Zhang, Robert E. Schaffert, Leon V. Kochian, Jurandir V. Magalhaes

**Affiliations:** 1 Embrapa Maize and Sorghum, Sete Lagoas, Minas Gerais, Brazil; 2 Institute for Genomic Diversity, Cornell University, Ithaca, New York, United States of America; 3 Robert W. Holley Center for Agriculture and Health, U.S. Department of Agriculture – Agricultural Research Service, Cornell University, Ithaca, New York, United States of America; Kansas State University, United States of America

## Abstract

Root damage caused by aluminum (Al) toxicity is a major cause of grain yield reduction on acid soils, which are prevalent in tropical and subtropical regions of the world where food security is most tenuous. In sorghum, Al tolerance is conferred by *SbMATE*, an Al-activated root citrate efflux transporter that underlies the major Al tolerance locus, *Alt_SB_*, on sorghum chromosome 3. We used association mapping to gain insights into the origin and evolution of Al tolerance in sorghum and to detect functional variants amenable to allele mining applications. Linkage disequilibrium across the *Alt_SB_* locus decreased much faster than in previous reports in sorghum, and reached basal levels at approximately 1000 bp. Accordingly, intra-locus recombination events were found to be extensive. SNPs and indels highly associated with Al tolerance showed a narrow frequency range, between 0.06 and 0.1, suggesting a rather recent origin of Al tolerance mutations within *Alt_SB_*. A haplotype network analysis suggested a single geographic and racial origin of causative mutations in primordial guinea domesticates in West Africa. Al tolerance assessment in accessions harboring recombinant haplotypes suggests that causative polymorphisms are localized to a ∼6 kb region including intronic polymorphisms and a transposon (MITE) insertion, whose size variation has been shown to be positively correlated with Al tolerance. The SNP with the strongest association signal, located in the second *SbMATE* intron, recovers 9 of the 14 highly Al tolerant accessions and 80% of all the Al tolerant and intermediately tolerant accessions in the association panel. Our results also demonstrate the pivotal importance of knowledge on the origin and evolution of Al tolerance mutations in molecular breeding applications. Allele mining strategies based on associated loci are expected to lead to the efficient identification, in diverse sorghum germplasm, of Al tolerant accessions able maintain grain yields under Al toxicity.

## Introduction

Among the various abiotic stresses that limit sorghum production, aluminum (Al) toxicity has been identified as one of the main breeding targets on acid soils [1], which are commonly found in tropical and subtropical regions where sorghum is intensively cultivated. As the primary result of Al toxicity is a damaged and stunted root system, water and nutrient acquisition are thereby compromised, leading to significant yield losses [2].

Because acid soils are widespread in the world [3], Al toxicity represents a major constraint for crop production worldwide, particularly in areas where food security still poses a significant challenge to human populations. For example, Al toxicity and phosphorus deficiency are major constraints for sorghum production in West Africa [4], [5]. In addition, yield reduction caused by drought stress, which is common in the region, worsens as roots intoxicated by Al are incapable of penetrating the deep, highly acidic soil layers to acquire water [6]. A potential threat to food security arises as sorghum and pearl millet are the main staple food crops in the West African Savannah zones [7]. In view of today’s challenge of feeding nine billion people in the near future, including the most economically disadvantaged [8], modern molecular strategies are needed [9].

Organic acids such as malate and citrate are released by Al-activated organic acid transporters located in the plasma membrane of cells in the root apex. Once in the rhizosphere, they form stable complexes with Al, thereby conferring Al tolerance [10]. Major Al tolerance genes belonging to the aluminum-activated malate transporter (ALMT) and multidrug and toxic compound efflux (MATE) families were first cloned in wheat (*TaALMT1*, [11]), sorghum (*SbMATE*, [12]) and barley (*HvAACT1*, [13]), and subsequently have been found to control Al tolerance in a number of other plant species (reviewed in [14]).

In sorghum, the major Al tolerance locus, *Alt_SB_*, was mapped to the end of sorghum chromosome 3 and explains 80% of the phenotypic variation in a mapping population derived from the Al-tolerant line, SC283, and the Al-sensitive line, BR007 [15]. An allelic series at the *Alt_SB_* locus was found to underlie highly contrasting phenotypes in sorghum. Evidence in support of other distinct Al tolerance genes was also found [16]. Subsequently, high resolution mapping localized *Alt_SB_* to a 24.6 kb region within which *SbMATE* (GenBank accession EF611342), a gene encoding an aluminum-activated citrate transporter belonging to the MATE family, was found to underlie the Al tolerance locus [12]. *SbMATE* is expressed in the roots of a tolerant near-isogenic line (NIL) in an aluminum-inducible fashion, with highest expression localized to the first centimeter of the root. The *SbMATE* coding region was completely monomorphic between the parental alleles. In conjunction with a significant positive correlation between Al tolerance and both citrate exudation and *SbMATE* expression, this suggests that polymorphisms in regulatory regions underlie the allelic effects at the *Alt_SB_* locus by modulating *SbMATE* expression. Polymorphisms in the *Alt_SB_* region included a variable Tourist-like miniature inverted repeat transposable element (MITE) insertion in the promoter region and SNPs and indels located in the second *SbMATE* intron and within two amplicons in the *SbMATE* 3′ region [12]. The size of the MITE insertion across different sorghum lines is positively correlated with Al tolerance, suggesting that this insertion harbors *cis*-acting elements that enhance *SbMATE* expression in Al tolerant genotypes. *Cis*-acting elements altering Al tolerance gene expression have been reported for *TaALMT1* in wheat [17], *HvAACT1* in barley [18] and for the *TaMATE1B* gene in wheat [19].

There is growing evidence that regulatory factors modulate the expression of Al tolerance genes. For example, in Arabidopsis, expression of both *AtALMT1* and *AtMATE* has been shown to be regulated by a C2H2-type zinc finger transcription factor, STOP1, which is also associated with tolerance to low pH [20], [21]. A homolog of STOP1, ART1, regulates the expression of a suite of genes related to Al tolerance in rice, including *STAR1* and STAR*2* [22], *Nrat1* [23], *OsALS1* (an ABC transporter involved in rice Al tolerance, [24]) and the MATE family member, *OsFRDL4* [25].

Using different donors, *Alt_SB_* was introgressed into a genetic background belonging to an Al sensitive line [26]. A varying reduction in both Al tolerance and *SbMATE* expression from parents to the derived NILs was observed and the NILs differed for *SbMATE* expression, suggesting that *SbMATE* expression is regulated at multiple levels. That is, these findings suggest that although *cis* effects are dominant in controlling *SbMATE* expression, the loss of functional *trans*-acting factors may lead to potentially strong genetic background effects depending on the donor allele, reducing both *SbMATE* expression and Al tolerance.

Population structure, which is common in sorghum [27], [28], [29], must be controlled in order to avoid false positives in association genetics research. We have previously reported that Al tolerance is a rare trait in sorghum and is not randomly distributed across the species diversity continuum [30]. That is, Caniato and colleagues found that 80% of the sorghum accessions were Al sensitive, 14% were identified as intermediately tolerant and only 6% were highly Al tolerant. Excluding breeding derivatives, only 5% of the entire panel was found to be highly tolerant to Al. Al tolerance is more prevalent in guinea and to lesser extent caudatum subpopulations, suggesting that causal mutations might have arisen in West Africa, after the guinea race differentiated from the primordial bicolor types.

In the present study, the panel described in [30] was used for association mapping, focusing on the 24.6 kb region where the *Alt_SB_* locus is located on sorghum chromosome 3. We observed a fast rate of LD decay in the region. Intra-locus recombination events were found to explain much of the haplotypic diversity observed for *Alt_SB_* and were instrumental in narrowing down the location of causal variants, which are likely located in a 6 kb region encompassing the MITE insertion and intronic polymorphisms. A haplotype network based on polymorphisms associated with Al tolerance suggests a single, recent origin of Al tolerance conferred by *Alt_SB_* in guinea sorghums from West Africa. The implication of these findings as a basis for allele mining strategies to identify Al tolerant accessions is discussed.

## Results

### Linkage Disequilibrium in the *Alt_SB_* Region

**Figure 1:**
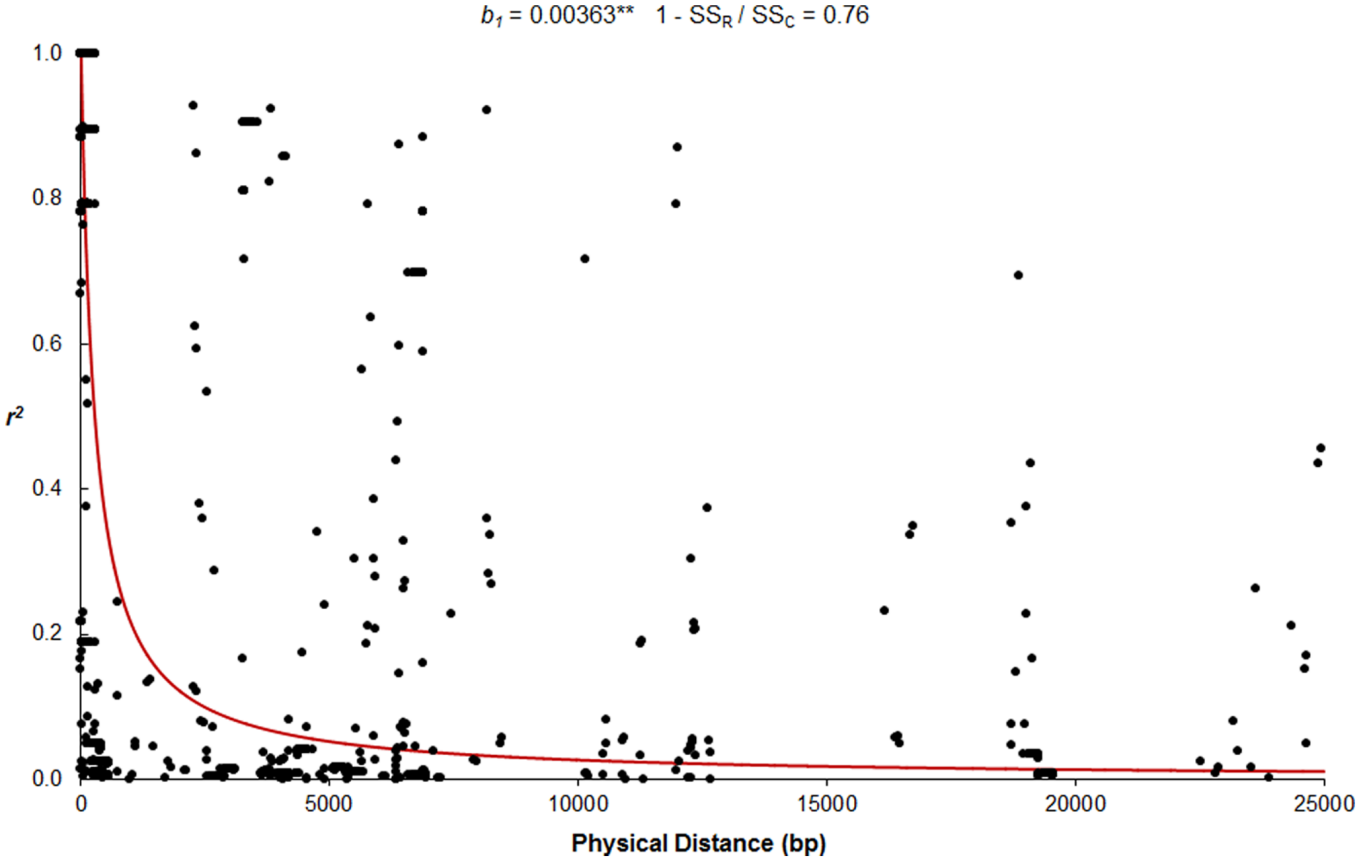
LD decay in the *Alt_SB_* region. In red is the prediction obtained by fitting a nonlinear regression model of the squared correlation of allele frequencies (*r^2^*) as a function of physical distance between pairs of loci based on the drift-recombination model [40]. The regression coefficient (*b_1_*, **p<0.0001) and the fraction of the total variance explained by the nonlinear model (1– SS_R_/SS_T_) are shown, where SS_R_ and SS_T_ are the sum of squares of error and total, respectively.

The LD decay model based on drift-recombination equilibrium explained approximately 76% of the variance in *r^2^* estimates. Figure 1 shows there is a steep decrease in *r^2^* estimates, which dropped to ∼0.2 and then close to zero for sites separated by ∼1 kb and ∼5 kb, respectively. An analysis of mean *r^2^* estimates and the respective standard deviations supports rapid LD decay in the *Alt_SB_* region (Table 1). Although LD decay was less pronounced based on *D’*, which is not sensitive to differences in allele frequencies [31], it was also significant using this statistic (regression coefficient, *b_1_* = 0.000028, *p*<0.0001). Based on *D’*, 85% of sites 1 kb apart but only 38% of sites more than 5 kb apart were in significant LD (*p*<0.05).

**Table 1:** Mean squared allele-frequency correlations (*r^2^*) and respective standard deviation across the *Alt_SB_* region.

Region (bp)	Number of sites	Mean *r^2^*	Standard Deviation
1–500	673	0.73	0.40
501–1024	51	0.02	0.04
1088–5079	289	0.14	0.30
5092–24934	418	0.11	0.22

### Association Model Fitting and Type I Error Control

**Figure 2:**
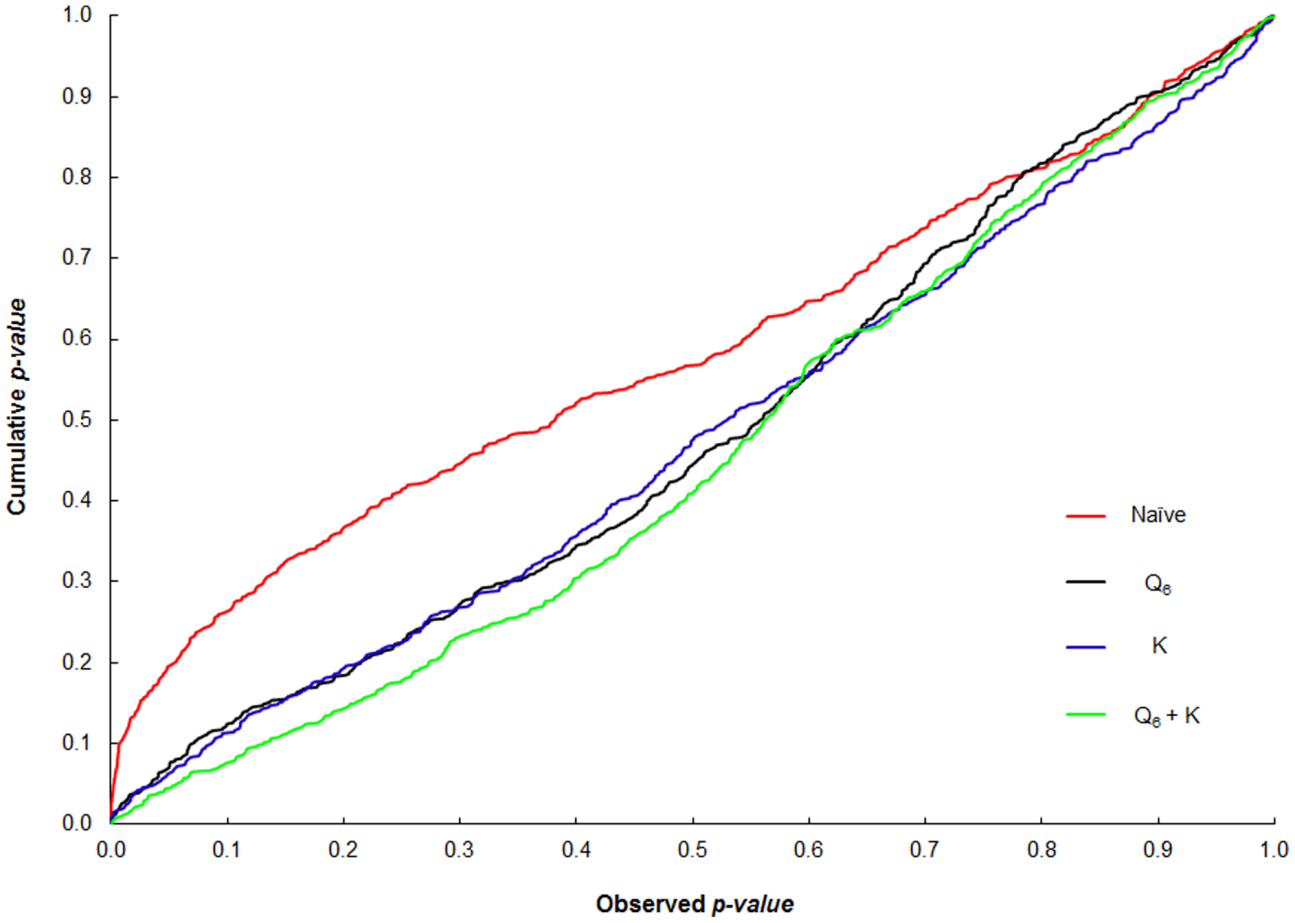
Model comparison for type I error control. Type I error distribution obtained with the naïve, Q_6_, K and Q_6_+ K models using 38 SSR loci and phenotypic traits related to Al tolerance. Under the expectation that the randomly distributed SSR loci are not associated with Al tolerance, models that properly control the type I error should show a uniform distribution of *p-*values along a diagonal line in the cumulative plot. Loci with MAF >0.1 were used.

For each tested model, the probability distribution under the null hypothesis was obtained by plotting the *p*-values resulting from association analysis against the cumulative *p*-values (Figure 2). A non-uniform distribution was found for the naïve, Q_6_ (six subpopulations) and K models in the cumulative plots, resulting in inflated type I error. Using a variety of approaches we have previously determined that six subpopulations result in a meaningful representation of the genetic diversity patterns in this sorghum association panel [30]. Nonetheless, here we also tested 4, 6, 8 and 12 subpopulations for type I error control. In agreement with our expectations, while the performance of the Q_4_ model in error control was reduced relative to Q_6_, no advantage was observed with higher subpopulation numbers (data not shown). Therefore, the most effective control of false positives was achieved with a mixed model that included six subpopulations combined with familial relatedness (Q_6_+K), which resulted in the fewest false positive associations among all tested models.

### Association Analysis Revealed Polymorphisms Associated with Al Tolerance

**Figure 3:**
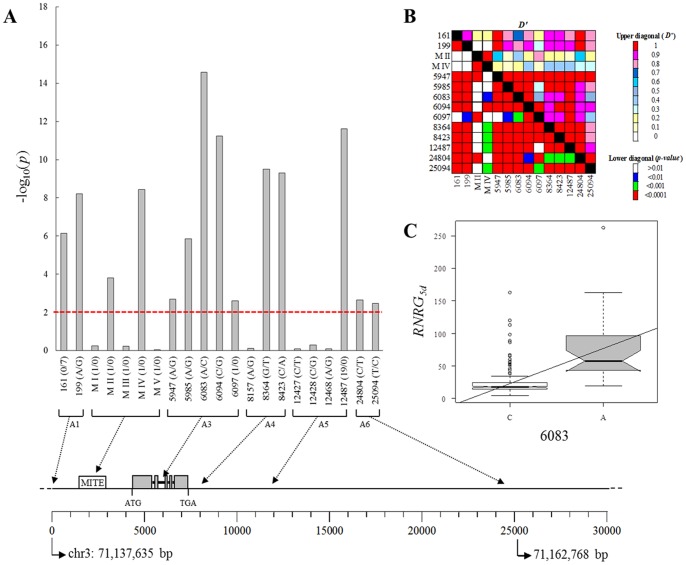
Association analysis for polymorphisms in the *Alt_SB_* region and Al tolerance. Association analysis with the Q_6_+ K model was performed with *RNRG_5d_*. (**A**) Statistical significance is expressed as –log_10_(*p*) and the *p*<0.01 threshold is represented by the red horizontal line. Polymorphisms are shown along the x-axis and are linked to the schematic below this graph which depicts their physical location in the 24.6 kb region where *SbMATE* (exons shown as gray boxes connected to black lines representing introns) was mapped on chromosome 3 (A1 to A5 depict amplicons harboring polymorphisms, Table S5). The corresponding physical positions in the sorghum genome are shown below the scale and were obtained by sequence similarity analysis (http://www.phytozome.net). The alleles at each loci are shown in the x-axis following the locus designation, with indels represented by the number of repeats, except for the MITE insertion, which was coded as described in the Material and Methods session. (**B**) Linkage disequilibrium expressed by pairwise *D’* estimates [65] among loci associated with Al tolerance. *p*-values obtained with the Fisher exact test are shown. (**C**) Allele substitution effect for the 6083 locus. The slope of the linear regression line indicates an allele substitution effect of 53.9% *RNRG_5d_* (*p*<2E-16).

Broad-sense heritability estimates for Al tolerance based on RNRG data exceeded 0.9. Using *p*<0.01 as a threshold, 14 of the 21 polymorphisms identified within the *Alt_SB_* region were found to be significantly associated with Al tolerance as represented by *RNRG_5d_* (Figure 3a), with five associated loci located within the second intron of *SbMATE*. As expected, associated loci were in general in LD (Figure 3b), preventing the unambiguous identification of causative polymorphism(s). The SNP locus with the strongest association was 6083 (−log_10_(*p*) ≅ 15) within the second *SbMATE* intron, explaining approximately 12% of the Al tolerance variation, with an allele substitution effect on Al inhibition of root growth (*RNRG_5d_*) of ∼54% (Figure 3c, Tables S1 and S2). At this locus, the allele associated with Al tolerance, A, was the least frequent allele (minor allele frequency, MAF = 0.10, Table S3 and S4). Loci with 8< −log_10_(*p*) <12 were 199, the MITE locus (MIV) in the promoter region, with a ∼43% effect on *RNRG_5d_*, marker 6094 in the second *SbMATE* intron and 8364, 8423 and 12487 downstream of *SbMATE*, which each increased *RNRG_5d_* by ∼60% (Table S2). Loci located on the edges of the *Alt_SB_* region up to the ∼12 kb position, such as 161, 199 and 12487, were still in significant LD with the loci located within or close to *SbMATE* and thus are still associated with Al tolerance. Associations became significantly weaker (−log_10_(*p*) ≅ 2) for polymorphisms located at physical positions relatively distant from *SbMATE*, near the 25 kb position in Figure 3a. Overall, this suggests that causal variants are located between loci 161 and 12487.

### Haplotype Diversity and the Fine Structure of the *Alt_SB_* Locus

**Figure 4:**
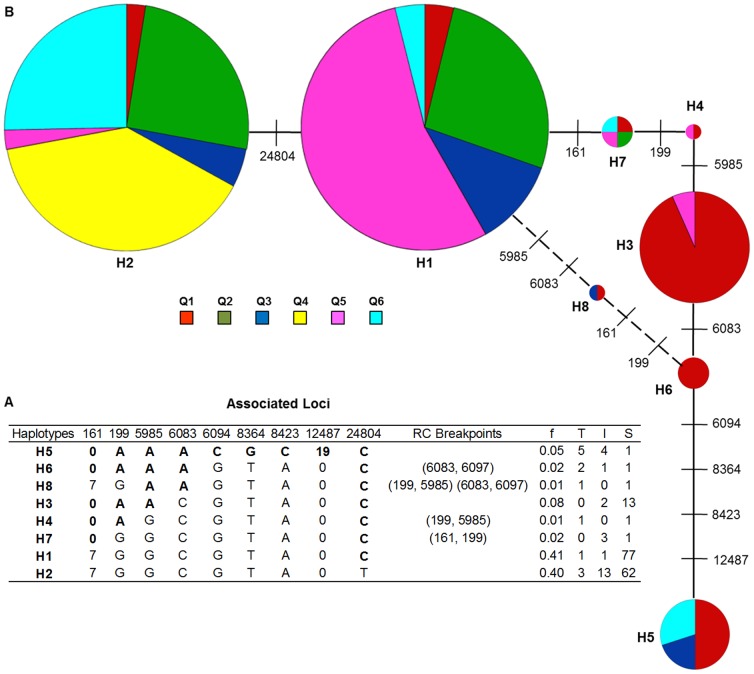
Relationship among *Alt_SB_* haplotypes. (**A**) Haplotypes with recombination (RC) breakpoints defined by the four-gamete test (two additional possible breakpoints between loci 6097/8364 and 12487/25094 were also detected). Alleles associated with Al tolerance (*RNRG_5d_*) are in bold (at the 161 and 12487 loci, 7/0 and 19/0 depict a 7 bp and a 19 bp indel). Haplotype frequency (f) and the number of Al tolerant (T, *RNRG_5d_* >80%) intermediate (I, 30%<*RNRG_5d_* <80%) and Al sensitive (S, *RNRG_5d_* <30%) accessions within each haplotype are shown. (**B**) *Alt_SB_* network based on nine sites associated with Al tolerance. The circle areas are proportional to haplotype frequencies except for H1and H2, whose areas were reduced by 2.5-fold due to their much higher frequencies. Colored areas are proportional to the number of accessions within each of the six subpopulations defined in [30]: Q1 (guinea accessions from western Africa and guinea margaritiferum accessions), Q2 (caudatum accessions from Africa and group of transplanted caudatum and durra accessions from Lake Chad region), Q3 (lines from the Embrapa collection and US), Q4 (kafir accessions from southern Africa), Q5 (durra accessions from central eastern Africa and from Asia; bicolor and caudatum accessions from Asia), Q6 (guinea accessions from southern Africa and Asia).

Consistent with the steep decrease in LD observed in the *Alt_SB_* region, the four gamete test revealed at least five likely recombination events in the region, with one intragenic recombination event detected in the second intron of *SbMATE* (between loci 6083 and 6097, Figure 4a). Because recombination may create homoplasy [32], introducing ambiguity into the relationships among *Alt_SB_* haplotypes, a haplotype network was built based on nine loci associated with Al tolerance, comprising eight different haplotypes (Figure 4b). This network shows the mutational relationships among the haplotypes, but it does not necessarily represent the mutational history, which is unknown. The two haplotypes with the highest frequency by far were H1 and H2, which differ only by a T(H2) ↔ C(H1) transition at the outer edge of the *Alt_SB_* region (locus 24804), with the C allele being present in all the other haplotypes.

Under neutrality, there is an expected relationship between haplotype frequency and haplotype age [33]. That is, the most common allele is likely to be the oldest with a probability equal to its frequency [34], [35]. Therefore, in view of their much higher frequency with respect to the other haplotypes, H1 and H2 appear to be the ancestral types in which Al tolerance mutations arose. Supporting this hypothesis is the high frequency of H1 in the subpopulation Q5 that includes the bicolor race, which is believed to be the ancestral morphological race from which the other sorghum races originated [36]. The only haplotype composed exclusively of alleles increasing Al tolerance was H5, which carries four alleles not found on any other haplotype. Interestingly, the putative ancestral haplotypes H2/H1 were formed exclusively by alleles associated with Al sensitivity, except for the C allele at the 24804 locus in H1. However, this locus showed only marginal association with Al tolerance and also had a negligible effect in *RNRG_5d_* (Table S2) indicating that it does not provide significant Al tolerance to the putative ancestral haplotype.

Incompatible splits depicted by loops in the network may represent events such as hybridization, horizontal gene transfer or recombination [37]. The low frequency haplotypes, H4, H6, H7 and H8, which are formed by alleles associated with both Al tolerance and Al sensitivity, are possibly the result of recombination events involving other haplotypes. Based on their higher frequencies and allelic constitution, with alternative alleles at all loci, H1 and H5 may have been involved in recombination events giving rise to H4, H6, H7 and H8. This is supported by the position of the recombination events detected by the four gamete test (see recombination breakpoints in H4, H6, H7 and H8 in Figure 4a).

Jointly considering H1 and H2, the percentage of Al tolerant and intermediate accessions harboring either haplotype was rather low (∼11%). In contrast, the vast majority of the accessions carrying the H5 haplotype, ∼90%, were either Al tolerant or intermediately tolerant. Significantly differently from the case for H1 and H2, H5 is present exclusively in subpopulations Q1, Q3 and Q6, which are composed primarily of guinea accessions with western (Q1) and southern African and Asian (Q6) origins, as well as breeding derivatives (Q3).

Interestingly, the A ↔ C transversion at the 6083 locus appears to correlate well with the occurrence of a high level of Al tolerance in sorghum. Considering the H5 haplotype and the putative recombinant haplotypes in the network loop, high Al tolerance was more frequently found in haplotypes carrying the A allele (eight Al tolerant accessions in H5, H6 and H8) rather than the C allele (only one Al tolerant accession in H3, H4 and H7) at the 6083 locus. Recombination was deemed important for the evolution of the β-globin region in humans [35]. Similarly, intra-locus recombination appears to be an important process underlying the haplotypic diversity for the sorghum Al tolerance locus, *Alt_SB_*.

### Allele Mining

In the context of allele mining, we then set out to investigate the power of different associated loci to recover Al tolerant and intermediate accessions in the association panel. Table 2 shows that loci combining high –log(*p*) for association with Al tolerance and lower MAFs, between 0.06 and 0.10, were the most efficient ones in recovering Al tolerant accessions in the panel. For example, the loci 6083 and 6094 in the second intron of *SbMATE*, and 8364, 8423 and 12487 downstream of its coding region, recovered between 79 to 86% of Al tolerant and intermediate accessions. Among those, the 6083 locus stands out as it recovers nine out of the 14 highly Al tolerant accessions. Complementarity between associated loci is compromised by the finding that loci recovering additional Al tolerant accessions, such as 6097 and 24804 showed rather high MAFs (0.32–0.39), undesirably co-selecting ∼80% of Al sensitive accessions.

**Table 2:** Recovery of Al tolerant and intermediate accessions by loci associated with Al tolerance.

									Al tolerant accessions													
Locus	Alleles	MAF	n	T	I	S	T+I (%)	S (%)	IS14351	IS21519	IS10801	SC566	SC283	SC175	CMS225	IS26554	IS23142	5DX	IS29691	IS25077	IS26457	Brandes
161	**0**/7	0.18	44	8	13	23	48	52	**+**	**+**	**+**		**+**		**+**	**+**				**+**	**+**	
199	**A**/G	0.17	41	8	11	22	46	54	**+**	**+**	**+**		**+**		**+**	**+**				**+**	**+**	
MII	**1**/0	0.40	103	3	15	85	17	83		**+**									**+**	**+**		**-**
MIV	**1**/0	0.11	25	5	11	9	64	36	**+**				**+**	**+**	**+**			**+**				**-**
5947	**G**/A	0.47	118	4	21	93	21	79	**+**	**+**	**+**	**+**	**+**		**+**	**+**				**+**	**+**	**+**
5985	**A**/G	0.19	47	9	12	26	45	55	**+**	**+**	**+**	**+**	**+**		**+**	**+**					**+**	**+**
6083	**A**/C	0.10	24	9	10	5	79	21	**+**	**+**	**+**	**+**	**+**		**+**	**+**					**+**	**+**
6094	**C**/G	0.07	16	6	7	3	81	19	**+**	**+**	**+**		**+**		**+**							**+**
6097	**0/**1	0.32	80	7	8	65	19	81	**+**	**+**	**+**	**+**	**+**	**+**	**+**	**+**	**+**	**+**	**+**		**+**	**+**
8364	**G**/T	0.06	14	5	6	3	79	21	**+**	**+**	**+**		**+**		**+**							
8423	**C**/A	0.06	16	6	7	3	81	19	**+**	**+**	**+**		**+**		**+**							**-**
12487	**19**/0	0.06	14	5	7	2	86	14	**+**	**+**	**+**		**+**		**+**							
24804	**T**/C	0.39	152	10	19	123	19	81	**+**	**+**	**+**	**+**	**+**		**+**	**+**	**+**			**+**	**+**	
25094	**T/**C	0.14	34	4	11	19	44	56	**+**		**+**		**+**		**+**							
Phenotypic	**–**	–	254	17	37	200	21	79														

In bold are the least frequent alleles, which are linked in coupling with Al tolerance except for loci with borderline association probabilities and/or small effects on *RNRG_5d_* (MII, 5947 and 24804). For each associated locus are shown the minor allele frequencies (MAF), the total number of accessions (n), the number of Al tolerant (T, *RNRG_5d_* >80%), intermediate (I, 30%<*RNRG_5d_* <80%) and sensitive (S, *RNRG_5d_* <30%) accessions. The proportions based on phenotypic selection (phenotypic) are shown at the bottom of the table. Given their genotypes, the Al tolerant accessions in the association panel marked with ‘**+**’ possess the Al tolerant *Alt_SB_* allele. The Al tolerant lines CMS226, CMS227 and 9929034, which are breeding derivatives from SC283 (Table S1 in [16]), were excluded from this analysis.

## Discussion

### Fast LD Decay in the *Alt_SB_* Region

Typically, LD decay estimates in sorghum are variable and range from 15–20 kb [38], to 50–100 kb [29] and as high as 150 kb [39]. Based on the drift-recombination model, LD in the *Alt_SB_* region based on *r^2^* dropped to ∼0.2 and close to zero for sites separated by 1 kb and 5 kb, respectively. Strikingly, this rather rapid rate of decay is more comparable to that in the outcrossing species, maize [40], [41], [42], than to that in previous reports on the largely self-pollinating sorghum. LD is the complex result of the history of recombination as well as the mutational history [43], which are profoundly affected by demographic factors among others. Interestingly, Lin and colleagues [44] screened 30,000 gametes to fine map the *Shattering1* (*Sh1*) gene to a 17 kb region on sorghum chromosome 1. In comparison, fine mapping of the *Alt_SB_* locus involved the screening of only 4,170 gametes, one seventh of the population size in the *Sh1* study, to resolve *SbMATE* into a 24.6 kb region [12]. Thus, for *Alt_SB_*, local factors appear to be dominant in determining the pattern of LD across the Al tolerance locus. It is possible that the *Alt_SB_* location towards the end of sorghum chromosome 3, which is enriched in gene content [45], is associated with local factors enhancing recombination. In maize, MacMullen and colleagues [46] observed the absence of loci with genome-wide effects on recombination, suggesting the importance of numerous but localized regions affecting recombination, which could be structural chromosome- or family-specific variants.

### Low Frequency Variants within the *Alt_SB_* Locus are Highly Associated with Al Tolerance

In the present study, we either sequenced or genotyped all polymorphisms previously detected between the parents of the mapping population used to positionally clone *SbMATE*. Causative polymorphisms are thus expected to be included among loci displaying statistically significant associations with Al tolerance. Association mapping is limited when the trait analyzed is correlated with population structure [47] as is the case of Al tolerance both in sorghum [30] and rice [48]. However, because the proportion of the phenotypic variance explained by population structure alone in our sorghum association panel was only 16% compared to 57% in rice, we expect that a substantial fraction of the phenotypic variance would still be available for capture by Quantitative Trait Nucleotides (QTNs), even in the presence of population membership cofactors in our mixed model. Indeed, 14 loci associated with Al tolerance were found in the *Alt_SB_* region, with 6083 showing the strongest association signal. Because multiple QTNs under LD may control Al tolerance conferred by the *Alt_SB_* locus, it is conceivable that 6083 reflects an indirect association with one or more causal polymorphism(s) in LD with this locus.

The proportions of Al tolerant and intermediate accessions in the association panel were 6 and 14%, respectively [30]. We observed that loci with extreme association probabilities (log_10_(*p*) >9) showed a rather narrow MAF range, between 0.06 and 0.11, with 6083 showing MAF = 0.10 (Table S3) and −log_10_(*p*) ≅ 15 for association with Al tolerance. Higher frequency common variants under LD with presumptive causal variants for the *Sh1* gene in sorghum led to synthetic association signals stronger than those of the causal variants [44]. Here, polymorphisms falling within the frequency interval for Al tolerant and intermediate accessions produced the strongest association signals. For example, although MAFs for loci in the more distant regions with respect to *SbMATE*, such as 161 (MAF = 0.18, Table S3) and 199 (0.17) in the 5′ region and 24804 (0.39) and 25094 (0.14) in the 3′ region were higher than that for 6083, the respective association signals were much lower. Because the Al tolerance range for intermediate accessions was rather broad (30%<*RNRG_5d_* <80%), it is likely that in addition to the Al tolerant accessions, only the fraction of the intermediate accessions that are most Al tolerant within that intermediate category contribute significantly to the association signals. As previously reported, the power to detect a QTL is expected to be the highest when the associated marker allele has a similar frequency to that of the QTL [49], [50].

### Causal Variants are Likely Localized in a 6 kb Region Encompassing a Variable MITE Insertion and Intronic Polymorphisms

The detection of extensive intra-locus recombination events (Figure 4b) provides support for fast dissipation of LD across the *Alt_SB_* region as revealed by fitting the drift-recombination model for LD decay. These recombinants may help to narrow down further the location of the causal variants affecting *SbMATE* expression.

Based on the proportion of Al tolerant and intermediate accessions within the Al tolerant haplotype, H5, and in the putatively recombinant haplotypes, the causative polymorphism(s) enhancing *SbMATE* expression and thus Al tolerance are likely located at or upstream of the 6083 locus within the second intron of *SbMATE*, including the MITE insertion in the promoter region. This leads to a reduction of the physical interval where causative mutations lie from previous 24.6 kb to only ∼6 kb. The location of the causal variants is supported by the observation that 8 of the 13 Al tolerant accessions in our haplotype network carried either the Al tolerant haplotype, H5, or recombinant haplotypes which retained either part (H8) or the whole (H6) region from H5 delimited by the loci, 161 and 6083, with all these accessions showing the H5 allele (A) at the 6083 locus. Among the accessions harboring H5 are the Al tolerant parent of the mapping population used to positionally clone *SbMATE*, SC283, and IS14351, which is the most Al tolerant accession in the panel [30], with both accessions showing high levels of *SbMATE* expression. Another highly Al tolerant line that shows high *SbMATE* expression is SC566 [30], which shows the recombinant haplotype, H8.

Of the remaining five Al tolerant accessions with the C allele at the 6083 locus, one accession carried the H4 haplotype, whereas one and three accessions carried the putative ancestral haplotypes, H1 and H2, respectively. In such a diverse panel, non-allelic heterogeneity in the form of different Al tolerance genes controlling distinct physiological mechanisms of Al tolerance may occur, as has been previously reported in rice for genes regulating panicle length [51]. In fact, we have previously reported both on allelic and non-allelic heterogeneity for Al tolerance based on the sorghum *Alt_SB_* locus [16, 26, 30). Our previous studies with NILs indicated extensive allelic heterogeneity in sorghum presumably arising from variations in *cis*-factors within *Alt_SB_* [26]. Therefore, the co-occurrence of weak *Alt_SB_* alleles showing low *SbMATE* expression in conjunction with other Al tolerance genes in the genetic background may account for the presence of Al tolerant accessions with the H1 and H2 haplotypes, which are formed by alleles linked in repulsion with Al tolerance. In fact, we have previously reported on non-allelic heterogeneity for Al tolerance in two accessions carrying the H2 haplotype, 5DX [16] and IS29691 [30]. Our data indicate that 5DX possesses a rather weak *Alt_SB_* allele and that the introgression of different Al tolerant gene(s) into the ‘DX’ genetic background is likely to account for its high levels of Al tolerance [16]. The *SbMATE* expression level in SC175, the remaining accession with the H2 haplotype, and in IS25077 that harbors H4, was approximately 4-fold smaller than that in the highly Al tolerant line, SC283 (see Figure 3 in [30] for data on IS25077 and Figure 3 in [26] for SC175), suggesting a similar situation in these lines to that observed in 5DX. Finally, although showing high *SbMATE* expression, marker-trait association with markers tightly linked to *Alt_SB_* in a backcross family derived from the single Al tolerant accession harboring the H1 haplotype, IS23142, was not significant (Figure 3 and Table S3, [30]). This could be due to the recessive mode of gene action for Al tolerance observed in this accession and/or the presence of other Al tolerance genes in its background. Therefore, our data suggest the presence of distinct, non *Alt_SB_*-based physiological mechanisms of Al tolerance in the putative ancestral haplotypes and in H4. Prevalence of accessions showing these mechanisms is expected in H1 and H2 due to the high frequency of these haplotypes in the association panel.

On the other hand, although the H5 haplotype was almost entirely composed of either Al tolerant or intermediate accessions, one Al-sensitive accession was found carrying this otherwise totally Al tolerant haplotype. One possible explanation is the significant occurrence of genetic background effects on Al tolerance controlled by *SbMATE* [26]. Accordingly, these background effects are the result of accessory loci acting in *trans*, without which *SbMATE* expression and Al tolerance can be dramatically reduced.

### A Possible Unified Origin of Al Tolerance Conferred by *Alt_SB_* in West African Guinea Types

The landrace accessions used in this study were chosen to provide representation of cultivated landrace sorghums from the whole world, with sampling based on race classification, latitude of origin, response to day length, and form of cultivation [52]. A core reference set including a large proportion of these accessions was found to capture ∼80% of the SSR alleles detected in a larger, 3367-member collection, indicating good representation of the global genetic diversity in sorghum [53]. This landrace collection has been recently characterized with over ∼265,000 SNP markers to investigate genomic patterns of diversification in sorghum [39].

The haplotypic diversity for *Alt_SB_* based on loci associated with Al tolerance provides insights into the origin of the mutations conferring Al tolerance in sorghum. The haplotype H5 was present exclusively in subpopulations Q1, Q3 and Q6, which are composed primarily of guinea accessions with western (Q1) and southern African and Asian (Q6) origins, as well as breeding derivatives (Q3).With a model selection procedure based on the Bayesian Information Criterion [54], we have previously shown that these three subpopulations are the most important ones in explaining the variation in Al tolerance across the association panel [30]. In addition, Q1 membership was the most prevalent in H5 accessions and was also frequent in haplotypes in the network loop, where recombination events involving H5 may have taken place.

In conjunction with the much higher frequency of the putative ancestral haplotypes H1/H2 compared to H5, the data presented here support a more recent origin of Al tolerance in the primary domestication center of the guinea race, in West Africa [36]. Given that differentiated haplotypes closely related to H5 but predominantly found in Q6 were not observed in the dataset, Al tolerant haplotypes in the secondary domestication center in South/East Africa are likely to have been transported from West Africa during the guinea radiation from the West to the southern domestication center of the guinea race [55], [56]. Therefore, the scenario described here suggests a single geographic and racial origin of Al tolerance mutations within *Alt_SB_* in primordial guinea domesticates in West Africa, with subsequent limited interracial spread of Al tolerance. This is consistent with our previous studies indicating non-random distribution of Al tolerance in the sorghum genetic diversity continuum [30].

### Analysis of the Power of Single SNPs versus *Alt_SB_* Haplotypes for Allele Mining in Sorghum

One important issue is whether single SNPs or haplotypes would be the most effective type of markers for allele mining based on *Alt_SB_*. Considering the associated loci with allele frequency closely matching that of Al tolerance (6083, 6094, 8364, 8423 and 12487), there would be no clear advantage in using haplotypes over the single SNPs for allele mining, as little or no complementation can be exploited in recovering Al tolerant accessions (note that the 6083 locus alone recovers most of the Al tolerant accessions in the panel). This is in line with the observation in barley that when the causal SNP is one of the genotyped markers, the power of single SNPs is superior to that of haplotypes [49]. The consequence of using loci with unmatched allele frequencies, such as 6097 which is only 14 bp from 6083, is the recovery of a large number of Al sensitive accessions, probably due to extensive recombination with the causal variant(s). However, allele mining based solely on 6083 would also recover ∼20% of the Al sensitive accessions in the panel, which is likely the result of genetic background effects reducing *SbMATE* expression as previously reported [26].

It is possible that multiple causal variants contribute to Al tolerance conferred by *Alt_SB_*. For example, the strong association signal observed for 6083 may reflect a role for the second *SbMATE* intron in enhancing gene expression. Introns are known to increase mRNA levels by acting as transcriptional enhancers, among other mechanisms [57], [58], [59]. In addition, here we show that the MITE insertion region in the *SbMATE* promoter is associated with Al tolerance. This result suggests a role for the transposon insertion in enhancing *SbMATE* expression, as has been observed in wheat Al tolerance involving *TaMATE1B* [19]. We are currently working to identify both the *cis* factors enhancing *SbMATE* expression and the *trans*-acting factors involved in genetic background effects.

The associated loci reported in this study are now available for high throughput germplasm screening through the Integrated Breeding Platform from the Generation Challenge Programme (https://www.integratedbreeding.net/). Using this Platform with the data presented here should allow us to build a molecular pipeline to increase yield stability for sorghum cultivated on acidic, Al toxic soils.

## Materials and Methods

### Plant Material

A set of 209 accessions from the landrace collection described in [52] and an additional 45 inbred lines formed the association panel that was used in this study. The landrace collection is representative of the genetic diversity present in cultivated sorghum [52], whereas the inbred lines are frequently used in breeding programs in the US and Brazil, including highly Al tolerant donors [16].

### Assessment of Al Tolerance in Nutrient Solution

Al tolerance data collection was undertaken previously [30]. A total of 254 sorghum accessions were evaluated in nutrient solution containing {0} or {27} µM Al^3+^. Values inside brackets indicate Al^3+^ activity, which was estimated with the speciation software program, GEOCHEM-EZ [60], [61].

Seeds of each genotype were germinated for four days and seedlings were transferred to containers with nutrient solution lacking Al at pH 4.0. After 24 h of acclimation, the initial length of each seedling’s primary root growing in control solution (*ilc*) was measured. The solution was then replaced with nutrient solution of identical composition but containing either no Al or {27} µM Al^3+^ supplied as AlK(SO_4_)_2_.12H_2_O. Final root lengths under Al treatment (*flAl*) or in control solution (*flc*) were obtained after three and five days of exposure to Al. For each inbred line, *r*elative percent values of *n*et *r*oot *g*rowth inhibition after three (*RNRG_3d_*) and five (*RNRG_5d_*) days of Al exposure were estimated by dividing the net root growth under Al treatment (*flAl*–*ilc*) by the net root growth without Al (*flc*–*ilc*). We adopted here the same Al tolerance classification described previously [30] for the sorghum accessions: Al sensitive (*RNRG_5d_* <30%), intermediately tolerant (30%<*RNRG_5d_* <80%, designated intermediate) and Al tolerant (*RNRG_5d_* >80%).

### Candidate Quantitative Trait Nucleotides (QTNs)

We previously sequenced the entire 24.6 kb *Alt_SB_* region in the Al tolerant and sensitive parents, SC283 and BR007, and identified candidate QTNs (depicted in Figure 1d in [12]). For the association analysis conducted here, we sequenced six amplicons spanning the QTN regions including a T↔A transversion in the first exon of *SbMATE* [26]. The genomic structure for the MITE insertion was previously obtained in four sorghum genotypes (Figure 3e and Supplementary Figure 3 in [12]). In the present study, the MITE insertion was genotyped in the association panel as five biallelic (presence/absence) loci. The four previously detected MITE alleles were designated MI (456 bp), MII (1,184 bp), MIII (1,514 bp), MIV (1,912). An additional 2,280 bp allele detected only in the association panel was designated MV.

### PCR and DNA Sequencing

Leaf tissues from three plants of each accession were used for DNA isolation according to [62]. Amplifications were carried out in a reaction volume of 20 µL that contained 30 ng of genomic DNA, 10X polymerase chain reaction buffer containing 0.5 mM dNTP, 4 mM MgCl_2_, 10 pmol of each primer, 5% of dimethyl sulfoxide (DMSO) and 1 U of Taq DNA polymerase (Phoneutria, Belo Horizonte, MG), following the amplification conditions described in Table S5 that also contains the sequences for the primers used in this study. PCR products were treated with 0.6 U of shrimp alkaline phosphatase (SAP, SB Corporation, Cleveland, OH) and 1.5 U of EXO I (USB Corporation, Cleveland, OH) in a reaction volume of 10 µL, which contained 6 µL of PCR reactions, 0.4 µL 10X SAP buffer (USB Corporation, Clevelend, OH). Digestion proceeded with incubation at 37°C for 30 min followed by 80°C for 10 min for enzyme inactivation. Sequencing reactions were carried out in a 10 µL reaction volume which contained 5 µL of digested PCR products, 2 µL of Big Dye V3.1 (Applied Biosystems, Forter City, CA), 2 µL of 5X buffer (Applied Biosystems, Forter City, CA) and 5 pmol of each primer. Sequencing reactions proceeded at 96°C for 4 min, 30 cycles at 96°C for 10 sec, 50°C for 5 sec and 60°C for 4 min. Sequencing reactions were analyzed on a ABI3100 sequencer (Applied Biosystems, Foster City, CA). Sequences were aligned and manually edited using the software SEQMAN (DNAstar, Madison, WI). MITE polymorphisms were scored on 1% (w/v) agarose gels.

### Analysis of Population Structure

Population structure (Q) was previously estimated based on 38 SSR loci which are evenly distributed across the sorghum genome, from a sorghum SSR kit ([53], http://sat.cirad.fr/sat/sorghum_SSR_kit/) developed within the Generation Challenge Programme (GCP, http://www.generationcp.org/). The complete description of the methods used for these analyses in addition to the SSR primer sequences and amplification conditions can be found in [30]. Briefly, the Bayesian cluster analysis as implemented in the software STRUCTURE [63], [64] was used to estimate the number of subpopulations based on the SSR data set. The admixture model with correlated allele frequencies was adopted, with a burn-in period of 100,000 and a run length of 1,000,000, with five independent replications for each k (number of subpopulations).

### Linkage Disequilibrium (LD)

LD between polymorphisms with minor allele frequency exceeding 0.05 was estimated using the standardized disequilibrium coefficient (*D*’, [65]) and squared allele-frequency correlations (*r^2^*, [66]) using the TASSEL software program (http://www.maizegenetics.net/bioinformatics/tasselindex.htm). Loci in significant LD based on D’ were defined with the Fisher’s exact test (*p*<0.05). The decay of LD with physical distance (bp) was estimated using nonlinear regression (PROC NLIN, SAS® software, SAS Institute Inc., Cary, NC, USA) based on the drift-recombination model described in [40]. Accordingly, the expected value of *r^2^* under drift-recombination equilibrium is E(*r^2^*) = 1/(1+*C*), where *C* = *4Nc* (*N* is effective population size and *c* is the recombination fraction between loci, [67]).

### Association Models

We tested three models to control for false positive associations (type I error) using the 38 randomly distributed SSR loci and various Al tolerance data: *RNRG_3d_*, *RNRG_5d_*, Visual Root Damage (*VRD)*, Induction of Root Growth (*IRG*) and Principal Components (PC) 1 and PC2, which are described in [30]). This marker density does not provide genome saturation within the low LD context in the association panel [29] and consequently, the chances of association with the phenotypic traits can be considered negligible. Thus, association analysis with these markers provides a null distribution to test the efficiency at which different models control for false positive associations. The naïve model, which does not account for familial relatedness or kinship, is **y = Aα+e**; the Q model, which accounts for population structure, is **y = Aα+Qν+e**; the K model, which accounts for familial relatedness or kinship, is expressed as **y = Aα+Zu+e**. In these models, **y** is a vector of phenotypic observations, **α** is a vector of fixed effects related to SNP effects (QTNs), **e** is a vector of residual effects, **ν** is a vector of fixed effects related to population structure and **u** is a vector of polygene background random effects related to familial relatedness. **A** and **Z** are the incidence matrices of 0s and 1s, relating **α** and **u,** respectively, to **y.** **Q** is the population membership assignment matrix obtained from the software, STRUCTURE relating **y** to **ν**. Finally, **y = Aα+Qν+Zu+e**, the unified Q+K mixed model that jointly accounts for population structure and familial relatedness [68] was fitted to the data. The variances of the random effects are expressed as **Var(u) = 2KVg** and **Var(e) = RV_R_,** where **K** is a 254×254 matrix based on the proportion of shared alleles values [69], obtained with the PowerMarker software [70], **R** is a 254×254 matrix with the off-diagonal elements being zero and the diagonal elements being the reciprocal of the number of observations for which each phenotypic data point was obtained, and **Vg and** **V_R_** are the genetic and residual variance, respectively.

### Haplotype Diversity and Network

A simplified haplotype network was constructed by maximum parsimony, using nine biallelic sites that were associated with Al tolerance, and omitting singleton haplotypes. The four-gamete test [71] was applied to identify possible recombination events.

## Supporting Information

Table S1 **Association statistics for loci in the** ***Alt_SB_*** **region.**
(DOC)

Table S2 **Allele substitution effects on** ***RNRG_5d_*** **(%) for loci associated with Al tolerance.**
(DOC)

Table S3 **Minor allele frequencies for loci in the** ***Alt_SB_*** **region.**
(DOC)

Table S4 **Complete genotypic and phenotypic dataset.**
(XLSX)

Table S5 **Primer sequences and amplification conditions.**
(DOC)
