# Supplementary material for: Association Mapping Provides Insights into the Origin and the Fine Structure of the Sorghum Aluminum Tolerance Locus, AltSB
Source: PLoS One. 2014 Jan 30;9(1):e87438. doi: 10.1371/journal.pone.0087438 (PMC3907521; doi:10.1371/journal.pone.0087438)
Supplement: Table S1 — Association statistics for loci in the AltSB region. (DOC) [file pone.0087438.s001.doc]

**Table S1. Association statistics for loci in the *Alt_SB_* region**

| Locus | Polymorphism |  | ***RNGR_3d_*** | ***RNRG_5d_*** |
| --- | --- | --- | --- | --- |
|  |  | F | 19.13 | 26.01 |
| 161 | **0**/7 | *P*-value | 1.89E-05 | 7.32E-07 |
|  |  | R^2^ (%) | 3.66 | 5.33 |
|  |  | F | 27.48 | 36.53 |
| 199 | **A**/G | *P*-value | 3.66E-07 | 6.22E-09 |
|  |  | R^2^ (%) | 4.89 | 7.03 |
|  |  | F | 0.00 | 0.32 |
| M I | **1**/0 | *P*-value | 0.9699 | 0.5728 |
|  |  | R^2^ (%) | 0.000298 | 0.073946 |
|  |  | F | 9.69 | 14.86 |
| M II | **1**/0 | *P*-value | 0.0021 | 1.53E-04 |
|  |  | R^2^ (%) | 1.93 | 3.23 |
|  |  | F | 0.17 | 0.27 |
| M III | **1**/0 | *P*-value | 0.678 | 0.6046 |
|  |  | R^2^ (%) | 0.036022 | 0.06232 |
|  |  | F | 32.82 | 37.83 |
| M IV | **1**/0 | *P*-value | 3.39E-08 | 3.72E-09 |
|  |  | R^2^ (%) | 5.94 | 7.47 |
|  |  | F | 0.08 | 0.02 |
| M V | **1**/0 | *P*-value | 0.7843 | 0.893 |
|  |  | R^2^ (%) | 0.015663 | 0.004208 |

| Locus | Polymorphism |  | **RNGR_3d_** | **RNRG_5d_** |
| --- | --- | --- | --- | --- |
|  |  | F | 7.07 | 9.73 |
| 5947 | **A**/G | *P*-value | 0.0084 | 0.002 |
|  |  | R^2^ (%) | 1.32 | 2.01 |
|  |  | F | 15.10 | 24.54 |
| 5985 | **A**/G | *P*-value | 1.33E-04 | 1.39E-06 |
|  |  | R^2^ (%) | 2.71 | 4.76 |
|  |  | F | 46.69 | 71.72 |
| 6083 | **A**/C | *P*-value | 7.12E-11 | 2.76E-15 |
|  |  | R^2^ (%) | 7.43 | 11.77 |
|  |  | F | 36.04 | 52.63 |
| 6094 | **C**/G | *P*-value | 7.31E-09 | 5.88E-12 |
|  |  | R^2^ (%) | 5.96 | 9.21 |
|  |  | F | 9.26 | 9.37 |
| 6097 | **1**/0 | *P*-value | 0.0026 | 0.0025 |
|  |  | R^2^ (%) | 1.7 | 1.93 |
|  |  | F | 0.06 | 0.10 |
| 8157 | **A**/G | *P*-value | 0.8079 | 0.752 |
|  |  | R^2^ (%) | 0.011274 | 0.021537 |
|  |  | F | 33.38 | 43.36 |
| 8364 | **G**/T | *P*-value | 2.48E-08 | 3.13E-10 |
|  |  | R^2^ (%) | 5.75 | 8.1 |
|  |  | F | 34.68 | 42.54 |
| 8423 | **C**/A | *P*-value | 1.53E-08 | 5.12E-10 |
|  |  | R^2^ (%) | 6.27 | 8.5 |

| Locus | Polymorphism |  | **RNGR_3d_** | **RNRG_5d_** |
| --- | --- | --- | --- | --- |
|  |  | F | 1.00 | 0.06 |
| 12427 | **C**/T | *P*-value | 0.3194 | 0.8068 |
|  |  | R^2^ (%) | 0.2 | 0.0133 |
|  |  | F | 0.71 | 0.43 |
| 12428 | **C**/G | *P*-value | 0.3991 | 0.5103 |
|  |  | R^2^ (%) | 0.14 | 0.096286 |
|  |  | F | 1.08 | 0.06 |
| 12468 | **A**/G | *P*-value | 0.2993 | 0.8081 |
|  |  | R^2^ (%) | 0.21 | 0.013034 |
|  |  | F | 37.28 | 54.79 |
| 12487 | **19**/0 | *P*-value | 4.25E-09 | 2.43E-12 |
|  |  | R^2^ (%) | 6.17 | 9.55 |
|  |  | F | 7.94 | 9.60 |
| 24804 | **C**/T | *P*-value | 0.0052 | 0.0022 |
|  |  | R^2^ (%) | 1.47 | 1.98 |
|  |  | F | 5.78 | 8.81 |
| 25094 | **T**/C | *P*-value | 0.017 | 0.0033 |
|  |  | R^2^ (%) | 1.11 | 1.88 |
