# Supplementary material for: Association Mapping Provides Insights into the Origin and the Fine Structure of the Sorghum Aluminum Tolerance Locus, AltSB
Source: PLoS One. 2014 Jan 30;9(1):e87438. doi: 10.1371/journal.pone.0087438 (PMC3907521; doi:10.1371/journal.pone.0087438)
Supplement: Table S2 — Allele substitution effects on RNRG5d (%) for loci associated with Al tolerance. (DOC) [file pone.0087438.s002.doc]

**Table S2. Allele substitution effects on *RNRG_5d_*(%) for loci associated with Al tolerance**

| Locus | Alleles | RNRG_5d_ (%) | Allele Substitution Effect (%*RNRG_5d_*) |
| --- | --- | --- | --- |
| 161 | 7 | 22.7 ± 19.3 |  |
|  | 0 | 53.5 ± 50.1 | 30.8 |
| 199 | G | 22.9 ± 19.3 |  |
|  | A | 55.2 ± 51.1 | 32.3 |
| M II | 0 | 32.8 ± 33.8 |  |
|  | 1 | 24.2 ± 18.1 | 8.6 |
| M IV | 0 | 23.9 ± 20.2 |  |
|  | 1 | 66.5 ± 56.7 | 42.6 |
| 5947 | G | 26.5 ± 21.9 |  |
|  | A | 30.3 ± 35.2 | 3.8 |
| 5985 | G | 22.8 ± 19.3 |  |
|  | A | 51.9 ± 48.4 | 29.1 |
| 6083 | C | 22.9 ± 18.5 |  |
|  | A | 76.8 ± 55.5 | 53.9 |
| 6094 | G | 24.2 ± 20.2 |  |
|  | C | 84.4 ± 62.3 | 60.2 |
| 6097 | 0 | 33.2 ± 41.3 |  |
|  | 1 | 26.2 ± 21.8 | 7.0 |
| 8364 | T | 24.2 ± 20.4 |  |
|  | G | 85.1 ± 66.2 | 60.9 |
| 8423 | A | 23.7 ± 20.4 |  |
|  | C | 85.1 ± 66.2 | 61.4 |
| 12487 | 0 | 24.3 ± 20.4 |  |
|  | 19 | 84.8 ± 65.2 | 60.5 |
| 24804 | T | 26.7 ± 19.6 |  |
|  | C | 29.6 ± 34.6 | 2.9 |
| 25094 | C | 25.1 ± 22.3 |  |
|  | T | 47.9 ± 49.5 | 22.8 |
