# Supplementary material for: Association Mapping Provides Insights into the Origin and the Fine Structure of the Sorghum Aluminum Tolerance Locus, AltSB
Source: PLoS One. 2014 Jan 30;9(1):e87438. doi: 10.1371/journal.pone.0087438 (PMC3907521; doi:10.1371/journal.pone.0087438)
Supplement: Table S3 — Minor allele frequencies for loci in the AltSB region. (DOC) [file pone.0087438.s003.doc]

**Table S3: Minor allele frequencies (MAF) for loci in the *Alt_SB_* region**

| Locus | Alleles | MAF |
| --- | --- | --- |
| 161 | (**0**/7) | 0.18 |
| 199 | (**A**/G) | 0.17 |
| MI | (1/0) | 0.21 |
| MII | (**1**/0) | 0.40 |
| MIII | (**1**/0) | 0.13 |
| MIV | (**1**/0) | 0.11 |
| MV | (**1**/0) | 0.06 |
| 5947 | (**G**/A) | 0.46 |
| 5985 | (**A**/G) | 0.19 |
| 6083 | (**A**/C) | 0.10 |
| 6094 | (**C**/G) | 0.07 |
| 6097 | (**0**/1) | 0.32 |
| 8157 | (**G**/A) | 0.23 |
| 8364 | (**G**/T) | 0.06 |
| 8423 | (**C**/A) | 0.06 |
| 12427 | (**T**/C) | 0.23 |
| 12428 | (**G**/C) | 0.07 |
| 12468 | (**G**/A) | 0.24 |
| 12487 | (**19**/1) | 0.06 |
| 24804 | (**T**/C) | 0.39 |
| 25094 | (**T**/C) | 0.14 |

The least frequent allele at each locus is marked in bold.
