# Supplementary material for: Association Mapping Provides Insights into the Origin and the Fine Structure of the Sorghum Aluminum Tolerance Locus, AltSB
Source: PLoS One. 2014 Jan 30;9(1):e87438. doi: 10.1371/journal.pone.0087438 (PMC3907521; doi:10.1371/journal.pone.0087438)
Supplement: Table S5 — Primer sequences and amplification conditions. (DOC) [file pone.0087438.s005.doc]

**Table S5: Primers sequences and amplification conditions**

| Primer Name | Genomic region | Position (bp) | Primer sequence 5’- 3’ |
| --- | --- | --- | --- |
| MJ6 | Amplicon 1 (A1) | 0 | GCCGAGCGGAGGAGGAC |
| MJ3 |  | 461 | TCGGCTGTCGGCTCCCTAAACTCA |
| MJ26 | MITE | 1,850 | ACACCGCCTTTTCAGTTTTTACG |
| MJ23 |  | 2,934 | CAGCGAATAATACTTTTAGTCATA |
| JL52 | *SbMATE_exon1 (A2:E1)* | 4,614 | AACAAGTGGCCAAGTGGGTGATCA |
| JL51 |  | 5,617 | GGCACGCACAGGCACAGTAACTTA |
| JL55 | *SbMATE_intron2 (A3:I2)* | 5,725 | GCCCGCGCTGCGCTACCTGA |
| JL56 |  | 6,439 | ATCCGAGGAAGCGCCGGAAT |
| M18 | Amplicon 4 (A4) | 8,071 | ACTCTGCGGTTTATTCGGATGGAT |
| M17 |  | 8,732 | ACCCGGAGCGTTCTTCTTG |
| JL237 | Amplicon 5 (A5) | 12,285 | GCAGATTCTTTGCCAGGTGT |
| JL238 |  | 13,009 | CAGTAGCAGGCATGATGTCG |
| JL225 | Amplicon 6 (A6) | 24,607 | TCTGGGCGGAATACAAGGTT |
| JL226 |  | 25,159 | TGGGAGTTCATGGTGGGTAAAA |

MJ3/MJ6: 95˚C – 2 min; 35 cycles: 96˚C – 10 sec, 58˚C – 10 sec, 72˚C – 30 sec; 72˚C – 5 min.

MJ23/MJ26: 95˚C – 2 min; 30 cycles: 94˚C – 60 sec, 55˚C – 60 sec, 72˚C – 2 min; 72˚C – 10 min.

JL51/JL52: 95˚C – 2 min; 35 cycles: 94˚C – 30 sec, 60˚C – 30 sec, 72˚C – 90 sec; 72˚C – 5 min.

JL55/JL56: 95˚C – 2 min; 35 cycles: 94˚C – 30 sec, 55˚C – 30 sec, 72˚C – 60 sec; 72˚C – 5 min.

MJ17/MJ18: 95˚C – 2 min; 30 cycles: 94˚C – 60 sec, 57˚C – 60 sec, 72˚C – 90 sec; 72˚C – 10 min

JL237/JL238: 95˚C – 2 min; 35 cycles: 94˚C – 30 sec, 55˚C – 30 sec, 72˚C – 60 sec; 72˚C – 5 min.

JL225/JL226: 95˚C – 2 min; 35 cycles: 94˚C – 30msec, 55˚C – 30 sec, 72˚C – 60 sec; 72˚C – 5 min.
